# Co-Registering Kinematics and Evoked Related Potentials during Visually Guided Reach-to-Grasp Movements

**DOI:** 10.1371/journal.pone.0065508

**Published:** 2013-06-03

**Authors:** Teresa De Sanctis, Vincenza Tarantino, Elisa Straulino, Chiara Begliomini, Umberto Castiello

**Affiliations:** Department of General Psychology, University of Padua, Padua, Italy; University of Rome, Italy

## Abstract

**Background:**

In non-human primates grasp-related sensorimotor transformations are accomplished in a circuit involving the anterior intraparietal sulcus (area AIP) and both the ventral and the dorsal sectors of the premotor cortex (vPMC and dPMC, respectively). Although a human homologue of such a circuit has been identified, the time course of activation of these cortical areas and how such activity relates to specific kinematic events has yet to be investigated.

**Methodology/Principal Findings:**

We combined kinematic and event-related potential techniques to explicitly test how activity within human grasping-related brain areas is modulated in time. Subjects were requested to reach towards and grasp either a small stimulus using a precision grip (i.e., the opposition of index finger and thumb) or a large stimulus using a whole hand grasp (i.e., the flexion of all digits around the stimulus). Results revealed a time course of activation starting at the level of parietal regions and continuing at the level of premotor regions. More specifically, we show that activity within these regions was tuned for specific grasps well before movement onset and this early tuning was carried over - as evidenced by kinematic analysis - during the preshaping period of the task.

**Conclusions/Significance:**

Data are discussed in terms of recent findings showing a marked differentiation across different grasps during premovement phases which was carried over into subsequent movement phases. These findings offer a substantial contribution to the current debate about the nature of the sensorimotor transformations underlying grasping. And provide new insights into the detailed movement information contained in the human preparatory activity for specific hand movements.

## Introduction

In our everyday life, we interact continually with objects. We reach for them, we grasp them, we manipulate them. All these actions are apparently very simple. Yet, this is not so. The mechanisms that underlie them are complex, and require multiple visuomotor transformations entailing the capacity to transform the visual features of the object in the appropriate hand configuration, and the capacity to execute and control hand and finger movements.

Motion analysis of grasping shows that the motor configuration that is formed by the hand in contact with the object represents the end result of a motor sequence that begins well ahead of the action of grasping itself [1]–[5]. The fingers begin to shape during transport of the hand towards the object. This process of preshaping first involves a progressive opening of the grip with straightening of the fingers, followed by a closure of the grip until it matches object size. The point in time where grip size is the largest (maximum grip size) is a clearly identifiable landmark that occurs well before the fingers come into contact with the object.

In neural terms, grasping behavior can be dissociated into separate reach and grip components (for review, see [6]–[11]). According to this view, computations regarding the grasp component occurs within a lateral parietofrontal circuit involving the anterior intraparietal area (AIP) and both the dorsal (PMd) and the ventral (PMv) premotor areas [12]–[17]. The general agreement is that the processes occurring in AIP constitute the initial step of the transformation leading from representation of objects to movement aimed at interacting with such objects [18], [19]. Evidence supporting this view comes from neurophysiological studies showing that the representation of 3D object features influences both the rostral sector of the ventral premotor cortex (area F5) and the ventro-rostral sector of the dorsal premotor area (area F2vr; [15]).

According to this model Area F5 plays a primary role in selecting the most appropriate type of grip on the basis of the object affordances provided by AIP to which it is reciprocally connected, thus activating a motor representation of the object. This motor representation is then supplied to area F2vr which keeps memory of it and combines it with visual information provided by cortical areas of the superior parietal lobe to continuously update the configuration and orientation of the hand as it approaches the to-be-grasped object. These properties suggest that F2vr neurons code the continuous activation of the object representation in motor terms, but that they are more dependent than F5 neurons on the visual information during actual grasp. With respect to the reach component, there is agreement that it is subserved by a more medial parieto-frontal circuit including the medial intraparietal area (mIP) termed as the parietal reach region (PRR), area V6A, and the dorsal premotor area F2 [20]–[24].

Human neuroimaging and transcranial magnetic stimulation (TMS) studies go in the same direction (for review see [6], [9], [25]–[30]). They showed the involvement of the anterior portion of the human AIP in grasping behavior [31]–[40] and they proposed human homologues of both the ventral and dorsal premotor cortices during grasping [38], [39], [41]. Whereas, reaching activates the medial intraparietal and the superior parieto-occipital cortex [42]–[45].

A point worth noting is that such dichotomic view has recently been questioned. Evidence from single-cell data [15], [46], [47] and lesion studies [48] suggests that areas V6a and F2 are also involved in managing specific aspects of grasping behavior such as grip posture and wrist orientation. Similarly, functional magnetic resonance imaging (fMRI) investigations reported grasping-related parieto-occipital and dorsal premotor cortex activations [38], [39], [42] which might be considered the possible human homologue for areas V6A and F2, respectively. Moreover, it is noteworthy that a recent neuroimaging study, based on the quantification of the modulation of the effective parieto-frontal connectivity, argues against the existence of dedicated circuits for reaching and grasping [49]. Rather, the authors suggest a differential level of effective connectivity in the AIP-PMv circuit depending on the type of grasped objects. Whereas grasping small objects is characterized by a high degree of on-line control requirement, grasping large objects led to an increased coupling in the so-called reaching circuit (V6A-PMd).

Complementary to these approaches, Evoked-Related Potentials (ERPs) measured by electroencephalography (EEG) provide a quantitative measure of the whole brain’s electrical activity, revealing the time course of brain activity modulations throughout reach-to-grasp movement from planning to execution. Wheaton and colleagues [50], [51] reported the involvement of parietal activity preceding that of the frontal areas in praxis hand movements. In this study, the authors compared motor potentials related to the generation of self-paced simple movements (i.e., thumb adduction) with motor potentials related to self-paced tool-use movements (e.g., hammer pantomime). Motor-related potential showed significant greater amplitude and earlier onset for more complex movements. Specifically, they observed that the motor-related potential in the posterior parietal cortex anticipated that in the frontal areas, and continued as the movement onset approached. They postulated that the complexity of the movement per se (e.g., multiple joint coordination) requires higher neural computation demand, which took place in the parietal lobe.

More recently, Bozzacchi et al. [52] defined the spatiotemporal activity of parietal and frontal areas in self-paced object-oriented actions. By examining motor-related potentials in planning reach-to-grasp movement, they clearly showed that parietal areas were involved in the early phase of planning. Such parietal activity started long before movement onset and was followed by a classical fronto-central component. The observed timing of parieto-frontal interaction in reach-to-grasp movements further confirmed previous evidence showing that parietal areas provide premotor areas with grasp-related information [49].

Another study [53] considering a precuing task found higher late Contingent Negative Variation (lCNV; [54]–[58]) amplitude over Cz and FC electrodes when the cue provided information regarding the type of grip to use and/or the level of force required to pull an object. Furthermore, whereas the force-related lCNV was more distributed over fronto-central electrodes, the grip-related lCNV was chiefly restricted to parietal and premotor areas. Aside from outlining the composite nature of the such ERP component in terms of high- and low-level planning processes, these findings confirmed that a functional parietal-premotor network is involved in the planning of grip [53].

To sum up, these studies suggest that in humans, like in monkeys, reach-to-grasp movements involve a large network of interconnected structures in the parietal and frontal lobes [8], [9], [59]. And, that this cortical network is differentially involved for the control of distinct aspects characterizing the planning and the control of reach-to-grasp movement. Nevertheless, how the neural control systems interact with the complex biomechanics of moving limbs - as to help us to identify the operational principles to look for in reach-to-grasp studies and, more in general, in motor control - remains an open question. In this respect, it is only through the use of converging techniques with different characteristics that we might fully understand how the human brain controls the grasping function [9]. What is so far lacking in the literature on cortical control of grasp in humans is a systematic documentation of the time course of neural activity and kinematical signals during performance of grasp. To fill this gap our study investigated ERPs with kinematical signals in order to provide deeper insights into the neuro-functional basis of grasping in humans. Participants were requested to perform a natural reach-to-grasp movement towards a visually available target object which could be either of a small size, requiring a precision grip movement (i.e., the opposition of the thumb and index finger) or of a larger size requiring a whole hand grasp (i.e., the opposition of the thumb with the other fingers) in order to be grasped. Differently from previous studies [50], we did not investigate ERPs evoked by a cue signaling specific object’s intrinsic features, but by the target stimulus itself. Such approach may allow to examine how information about an object’s geometric properties is transformed into specific motor programs more directly. We hypothesize that the ERP analysis may reveal the time course of activation of the differential cortical areas related to the planning, initiation and on-line control of reach-to-grasp movements and how such activity varies depending on grasp types. Kinematic analysis will provide an objective standard for parsing hand movements into distinct stages and for determining their temporal occurrence. Hand movements kinematics, acquired by means of a three-dimensional (3D) motion analysis system synchronized with the EEG recording system, will make possible the correlation across neural and kinematical temporal events.

## Materials and Methods

### Ethics Statement

The experimental procedures were approved by the Institutional Review Board at the University of Padua, and were in accordance with the Declaration of Helsinki (Sixth revision, 2008). All participants gave their informed written consent to participate in the study.

### Participants

Twenty-two students, recruited from the Faculty of Psychology at the University of Padua, took part in the study. They had a mean age of 23.68 years (SD = 2.49; range = 19.28; 11 females); they were all right handed, as measured by the Edinburgh Handedness Inventory [60], with normal or corrected to-normal vision, and without neurological or psychiatric pathologies.

### Apparatus and Procedures

**Figure 1:**
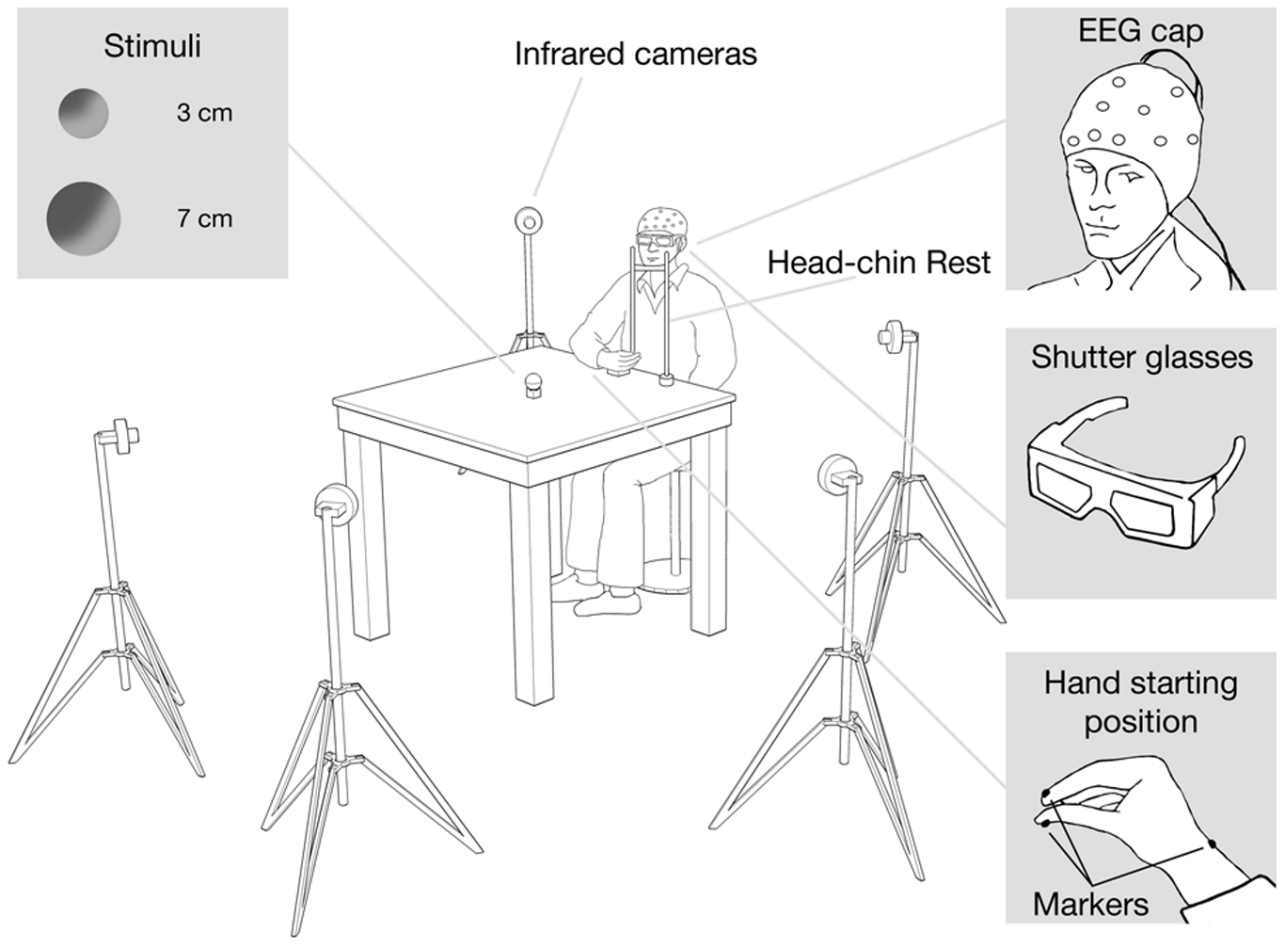
Experimental set-up.

**Figure 2:**
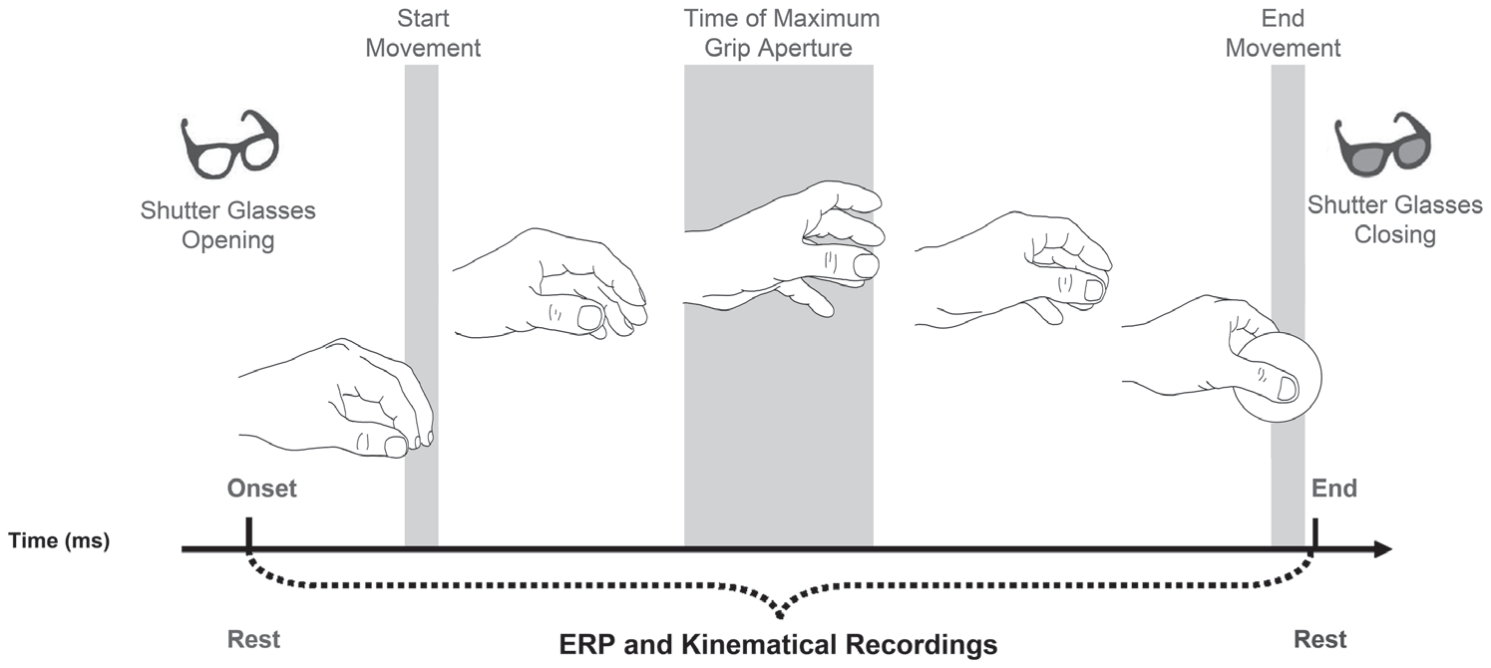
Hand choreography and type of recordings. Graphical representation of the choreography assumed by the hand during the movement and the timeline within which ERPs and kinematical data were acquired.

The participant was seated on a height adjustable chair so that the thorax pressed gently against the front edge of the table and the feet were supported. The position of the head was controlled by means of a head-chin-rest. A pressure sensitive starting switch was positioned 15 cm anterior to the mid-line of the participant’s thorax. With the hypothenar eminence of the right hand placed upon this switch, the starting position was slight shoulder flexion and 70–80° of internal rotation, 90° of elbow flexion, semipronation of the forearm, 5–10° wrist extension and opposition between the pads of the index finger and thumb. The experimental stimuli were either a small or a large wooden sphere (Figure 1). The small sphere was of diameter 3 cm whereas the large sphere was of diameter 7 cm. The stimulus was placed upon the working surface 30 cm directly in front of a pressure sensitive starting switch (Figure 1). Visual availability of the stimulus was controlled via Plato Liquid-crystal shutter glasses (translucent Technologies, Toronto, ON, Canada) worn by the participant throughout the test (Figure 1). Under computer control, the shutters change from translucent to transparent within 10 ms and return to translucent in 2 ms. All participants naturally adopted a precision grip (PG, opposition between the index finger and thumb) to grasp the small stimulus and whole hand prehension (WHP, all fingers opposing the thumb) to grasp the large stimulus. And they were requested to maintain their gaze fixed towards the stimulus location. There were two experimental conditions, a grasping large (GL) condition in which participants grasped the large stimulus adopting a WHP. And a grasping small (GS) condition in which participants grasped the small stimulus adopting a PG. During a training session task instructions were given to participants. The experimenter explained the task consisting in reaching towards and grasping the presented stimulus. Once the participant was comfortable with the task they performed a total of 80 trials, 40 for the GL and 40 for the GS conditions. The sequence of events was the following. At the start the shutter glasses were in a closed (opaque) state. At the time the shutter glasses opened (i.e., became translucent) the stimulus become visible and the participant was instructed to initiate the reach to grasp movement towards the stimulus. The shutter glasses remained open for the entire duration of the movement. Trials were administered in two blocks presented in a pseudorandom order. All failed trials were reintegrated and presented randomly later in the block. ERPs and kinematical recordings started at the time the shutters glasses became translucent (Figure 2).

#### Kinematical recording and data processing

Reflective passive markers (0.25 cm diameter) were attached to the following points of the reaching limb: (a) wrist - radial aspect of the distal styloid process of the radius; (b) index finger - radial side of the nail; and (c) thumb - ulnar side of the nail (Figure 1). Movements were recorded with the SMART system (BTS, Milan, Italy). This consisted of six infra-red cameras (sampling rate 200 Hz) inclined at an angle of 45 degrees to the vertical, and placed around the table (Figure 1). The calibrated working space was a parallelepiped (length 50 cm, breadth 50 cm, height 50 cm) from which the spatial error measured from stationary and moving stimuli was 0.4 mm. Coordinates of the markers were reconstructed with an accuracy of 1/3000 over the field of view and sent to a host computer. The SD of the reconstruction error was 1/3000 for the vertical (Y) axis and 1.4/3000 for the two horizontal (X and Z) axes. The SMART analyzer software package was used to assess the data. This gave a three-dimensional reconstruction of the marker positions. The data were then filtered using a finite impulse response (FIR) linear filter - transition band of 1 Hz (sharpening variable = 2; cutoff frequency 10 Hz). The reach component was assessed by analysing the trajectory and the velocity profile of the wrist marker. The manipulation component was assessed by analysing the trajectory of each of the hand markers, and the distance between these two markers. Reaction time was defined as the time interval between the opening of the crystal liquid lenses and the release of the start button upon which the hand was resting. Movement duration was calculated as the time between movement onset (defined as the time at which the wrist first began to move) and the end of the action (defined as the time when the fingers closed on the target and there were no further changes in the distance between the index finger and thumb). The period following this, whereby the stimulus was lifted, was not assessed. The dependent variables were (a) reaction time; (b) movement duration, (c) transport component parameters: time and amplitude of peak velocity of the wrist marker, and (c) grasp component parameters: time and amplitude of maximum grip aperture.

#### Electrophysiological recording and data processing

The electroencephalogram (EEG) was acquired by a portable amplifier system (SD-MRI, Micromed, Mogliano Veneto, Italy) from an array of 30 tin electrodes embedded in an elastic cap (ElectroCap International, Inc.) according to the 10–20 International System (AEEGS, 1991). The montage included the following scalp positions: Fp1,Fpz, Fp2, F7, F3, Fz, F4, F8, FC3, FCz, FC4, T3, C3, Cz, C4,FT7, FT8, T3, T8, T5, CP3, CPz, CP4, P3, Pz, P4, T6, TP7,TP8, O1, O2. All electrodes were referenced to linked-mastoids. The ground electrode was placed in AFz. Impedance of all electrodes was kept below 5 kΩ. The EEG signal were digitized at a sampling rate of 512 Hz (16 bit AD converter), and high-pass filtered at 0.15 Hz. Data processing was performed by BrainVision Analyzer 2 software (Brain Products GmbH, Gilching, Germany). Continuous EEG was off-line low-pass filtered at 30 Hz. Epochs were extracted separately for each of the two type of object stimuli (small, large), time-locked at the time the glasses were opened (i.e., stimulus appearance) and lasted 2000 ms. The considered time window encompassed the time at which the shutter glasses opened and the time at which the object was grasped (see Figure 2). Artifacts were corrected by means of Independent Component Analysis (ICA) applied on all epochs together, regardless of stimulus size. The ICA correction was performed by using a toolbox in the EEGLAB software (9.0.3.4b version; [59]). The ICA allows for the identification of the independent components in the segmented EEG signal by taking simultaneously into account frequency, timing and location on the scalp. This procedure helps in isolating artifactual components, such as blinks and head muscles’ contraction [61]–[63]. In addition, epochs containing amplitude deflection greater than ±75 µV was rejected for all the recorded channels prior to further analysis. The signal was then baseline-corrected against the mean voltage during the 200 ms prior to object appearance. Epochs containing erroneous movements were discarded. A total of 38.31 epochs (SD = 1.84) for each size condition were included within the statistical analyses. Based on visual inspection of grand average waveforms and amplitude scalp maps, the following ERP components were statistically analyzed: amplitude and latency of P300, namely the positive peak evoked 200–400 ms following stimulus appearance at occipital (O1, O2) and parietal (Pz) sites; amplitude and latency of N400, namely the negative peak occurring at 300–500 ms after object appearance at frontal (F3, Fz, F4), fronto-central (FC4, FCz, FC3), and central (C3, Cz, C4) sites; and mean amplitude of the sustained negativity observed in 400–800 and 1200–2000 time windows at frontal (F3, Fz, F4), fronto-central (FC4, FCz, FC3), central (C3, Cz, C4), and parietal (P3, Pz, P4) sites.

### Data Analysis

Mean values for reaction time, movement duration and each kinematical measure were compared between grasping conditions (small, large) by means of paired t-test. ERP components were analyzed by means of separate repeated measure ANOVAs (see ‘results’ section). The alpha level of significance was fixed at 0.05. Before running the analyses, we checked for all the main assumptions behind this statistical parametric model (i.e., normality and sphericity). Kolmogorov-Smirnov test revealed that the normality assumption was satisfied. In all ANOVAs, Mauchly test showed that the sphericity assumption was not violated. The effect size of ANOVA results was quantified by means of partial eta-square values (η^2^_p_). P-values of t-test and correlation results were corrected for multiple comparisons using a false discovery rate (FDR). Post-hoc comparisons of ANOVA were corrected by Bonferroni method. Correlation analyses by means of Pearson’s *r* coefficient were performed between kinematical and ERP measures as well as between movement duration and ERPs events.

## Results

### Reaction Time and Movement Duration

Reaction time did not differ between the GL and the GS conditions (517±137 vs 500±123 ms; p>0.05). However, movements towards the smaller stimulus had a longer duration than movements towards the larger stimulus [1141±164 vs 1114±196 ms; F (1,23) = 5.46, p<0.02; η^2^_ p_ = 0.41].

### Kinematics

The manipulation of object size had predictable effects on the reaching and the grasping component, respectively. In particular, the reach component was characterized by a bell-shaped wrist velocity profile with a single peak. The latency of this peak did not differ significantly with stimulus size (475±123 vs 476±130 ms). For the grasp component, there was a direct relationship between the size of the stimulus and the maximum opening of the hand en route to the target, and between the size of the object and the time taken to open the hand maximally. The maximum grip aperture occurred earlier [519±48 vs 582±61 ms; F (1,23) = 16.06, p<0.001; η^2^_ p_ = 0.46] and it was smaller [123±3 vs 91±2 mm; F(1,23) = 106.93, p<0.0001; η^2^_p_ = 0.58] for the GS than for the GL conditions.

### Evoked Related Potentials

**Figure 3:**
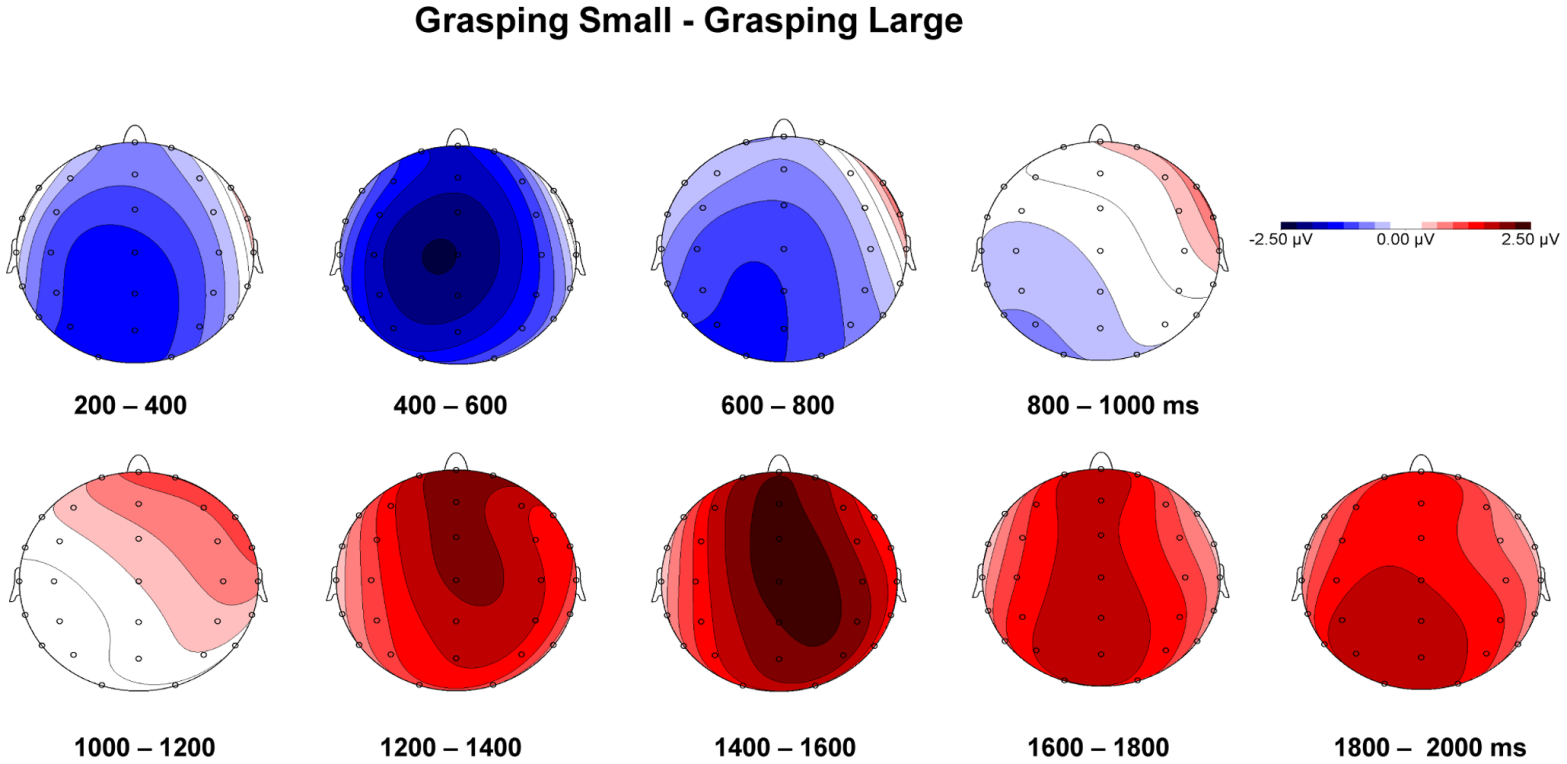
Grand-average ERP waveforms and amplitude scalp map of the P300 component. The plots in panel A show ERPs time-locked to goggles opening at parietal electrode sites in Grasping Large (blue line) and Grasping Small (red line). In panel B, the topographical distribution of the P300 component is represented. Differences between the two experimental conditions (Grasping Large– Grasping Small) were considered.

**Figure 4:**
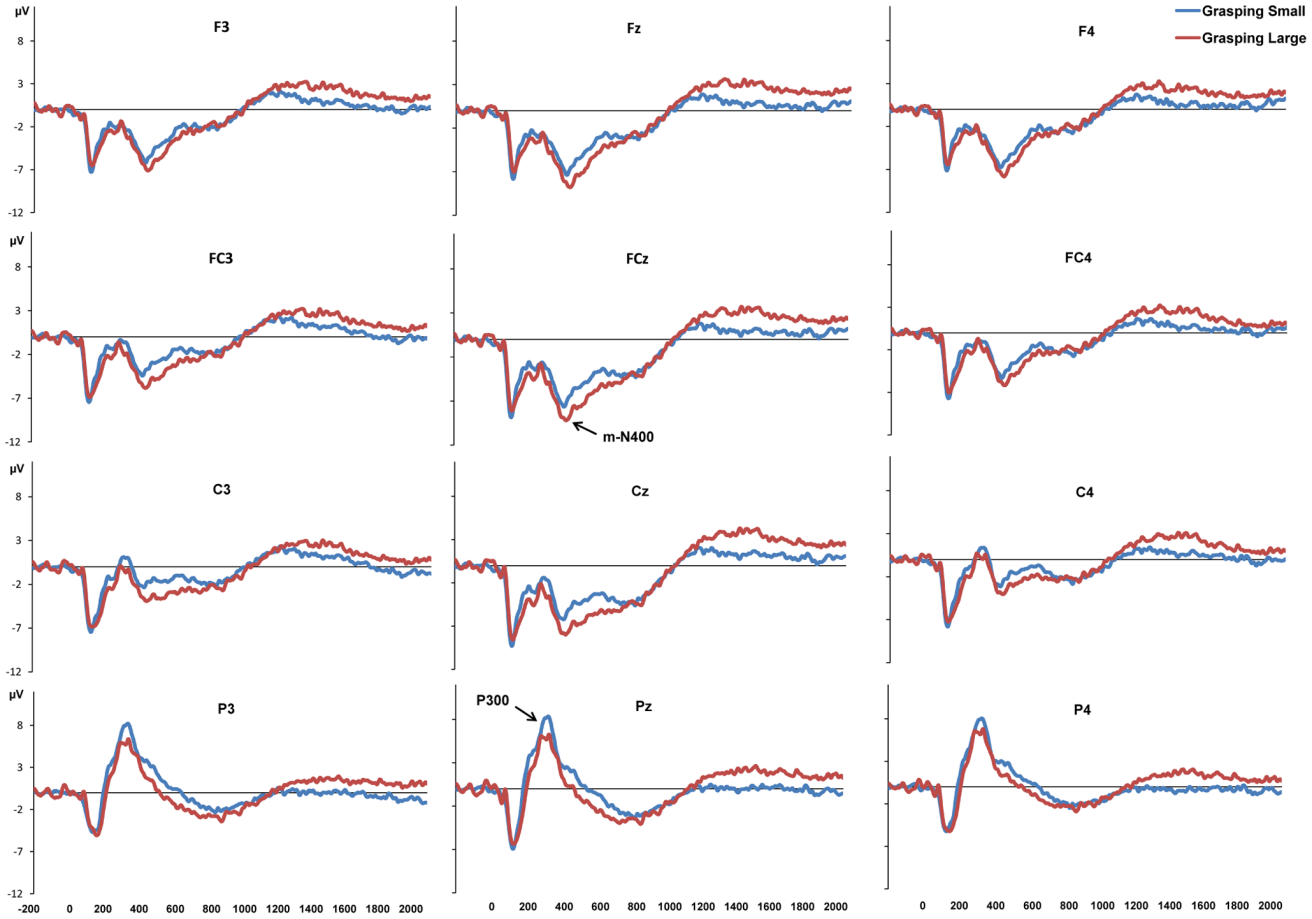
Grand-average ERP waveforms and difference amplitude scalp map of the m-N400 component. The plots in panel A show the ERPs time-locked to goggles opening at frontal, centro-frontal and central electrode sites for the Grasping Large (blue line) and the Grasping Small (red line) conditions. In panel B, the topographical distribution for the m-N400 component is represented. These plots represent the differences between the two experimental conditions (Grasping Small – Grasping Large).

ERP waveforms of grand-average, locked to glasses opening (i.e., object appearance), were characterized by an early negative peak at around 100 ms, more marked at parietal and central electrode sites, which showed similar amplitude and latency for the two grasping conditions. Then, differences in ERP amplitude between the two conditions become evident. Specifically, a positive peak at around 300 ms (P300), maximally expressed at parietal electrode sites, showed higher amplitude for the GL than for the GS conditions. Subsequently, a negative electrical activity, peaking at around 400 ms, evident at central and frontal electrode sites, and sustained for a time-window lasting from 400 to 800 ms, clearly showed higher amplitude for the GS than the GL conditions. The polarity, the temporal trend and the scalp distribution for such component suggests that this is likely linked to the motor component of action planning and to premotor areas, therefore we termed this component as motor-related N400 (m-N400). From 800 to about 1200 ms after object visual availability, a slow ERP deflection from negative to positive values at all electrode sites was found, which was characterized by a similar pattern for the two conditions (see Figures 3 and 4). Then, a sustained positivity was evident from 1200 to 2000 ms, which was higher for the GS than for the GL condition. A time window corresponding to the time at which the object was approached and contact points have to be optimized.

#### P300

Figure 3 depicts grand-average waveforms for the two grasping conditions at parietal sites P3, Pz, and P4, in which a P300 was evident. The amplitude and the latency of this component were analyzed by means of a 2 (stimulus size: small, large)×3 (electrode position: left, midline, right) repeated-measure ANOVA. The analysis revealed a main effect of stimulus size [F (1,21) = 5.98, p = 0.024; η^2^_p_ = 0.24]. A less positive amplitude for the GS than for the GL condition was revealed. No difference in peak latency between grasping conditions was found. Rather, electrode position significantly affected latency of the P300 component [F (2,20) = 4.22, p = 0.022; η^2^_p_ = 0.18]. Post-hoc comparisons revealed that P300 peak reached maximal amplitude earlier for the left and midline sites (P3 and Pz, respectively) compared to the right site P4 (p = 0.024 and p = 0.047, respectively). No significant differences between P3 and PZ were detected. The scalp map in Figure 3, showing the topography of differential amplitude (GL – GS), confirms that within the 300–350 ms time window ERPs were higher for the GL compared to the GS condition.

#### m-N400

Figure 4 illustrates grand-average waveforms for the two grasping movements at the following electrode positions: F3, Fz, F4, FC4, FCz, FC3, C3, Cz, C4. Amplitude and latency of the negative ERP deflection peaking at around 400 ms (m-N400) were analyzed by means of a 2 (stimulus size: small, large) × 3 (anterior-posterior electrode position: frontal, fronto-central, and central) × 3 (left-right electrode position: left, midline, right) repeated-measure ANOVA. This analysis yielded a main effect of stimulus size [F (1,21) = 7.18, p = 0.014; η^2^_p_ = 0.25], namely the m-N400 peak was found to reach higher amplitudes when participants were required to grasp the small compared to the large stimulus. A main effect of anterior-posterior electrode position was found [F (2,20) = 14.78, p<0.001; η^2^_p_ = 0.41]. Post-hoc comparisons revealed that, for both conditions, m-N400 amplitude was higher at frontal compared to fronto-central (p = 0.30) and central (p = 0.002) sites, and at fronto-central compared to central sites (p = 0.001). Furthermore, a main effect of left-right electrode position [F (2,20) = 33.07, p<0.001; η^2^_p_ = 0.61] showed that, for both grasping conditions, m-N400 amplitude was higher at midline compared to left (p<0.001) and right (p<0.001) sites. The post-hoc analysis of the stimulus size × anterior-posterior electrode position interaction [F (2,20) = 3.76, p = 0.031; η^2^_p_ = 0.15] revealed that for the GS condition the amplitude of the m-N400 did not differ between frontal and fronto-central sites (p = 0.205), meaning that the m-N400 was more equally distributed at frontal and fronto-central sites. Furthermore, the significant anterior-posterior × left-right electrode position interaction [F(4,18) = 18.27, p<0.001; η^2^_p_ = 0.46] revealed that for both grasping conditions, the m-N400 amplitude significantly increased from central to fronto-central to frontal sites only in left and right electrodes (all ps ≤0.040), whereas for midline electrodes it was equally larger. When considering latencies a main effect of stimulus size was found for them-N400 [F (1,21) = 8.65, p = 0.008; η^2^_p_ = 0.30]. For all the considered electrode sites the m-N400 reached the maximum values later for the GS than for the GL condition. In summary, the m-N400 showed higher amplitude and later latency for the GS than for the GL condition at all considered electrode sites. Specifically, the maximum peak value was reached at FCz (GS: MAmpl = −12.13 µV, MSE = 1.13; MLat = 429.97 ms, MSE = 16.82; GL: MAmpl = −10.11 µV, MSE = 1.10; MLat = 383.55 ms, MSE = 18.37). The differential scalp distribution for the m-N400 component is depicted in Figure 4, where it clearly appears that this component reached its maximal (negative) amplitude values for the GS condition at frontal and central midline electrode sites.

#### 400–800 ms

As shown in Figures 3 and 4, a sustained potential was observed from 400 to 800 ms at frontal, fronto-central, central, and parietal electrode sites (F3, Fz, F4, FC4, FCz, FC3, C3, Cz, C4, P3, Pz, P4). Mean ERP amplitude in this time window was analyzed. The 2 (object size) × 4 (anterior-posterior electrode position)×3 (left-right electrode position) ANOVA revealed that, as found for the m-N400 peak, such component showed an overall higher (more negative) mean ERP amplitude for the GS compared to the GL condition, in all frontal, fronto-central and central electrode sites [main effect of stimulus size: F (1,21) = 7.72, p = 0.011; η^2^_p_ = .27]. A significant main effect of anterior-posterior electrode position [F (2,20) = 46.69, p<0.001; η^2^_p_ = 0.69] revealed that, for both grasping conditions, larger ERP amplitude was observed at frontal and fronto-central sites compared to central and parietal positions (all ps <0.003). A significant main effect of left-right electrode position [F (2,20) = 31.50, p<0.001; η^2^_p_ = 0.60] showed that mean ERP amplitude within the 400–800 time-window was maximal at midline compared to both left and right sites (ps <0.001). As for the m-N400, the significant stimulus size × anterior-posterior electrode position interaction [F (2,20) = 4.92, p = 0.012; η^2^_p_ = 0.19] revealed that for the GS condition such sustained negativity was equally distributed between frontal and fronto-central sites (i.e., mean ERP amplitude between such sites did not differ, p = 0.625). The anterior-posterior × left-right electrode position interaction [F (4,18) = 13.76, p<0.001; η^2^_p_ = 0.40] revealed that, for both grasping conditions, ERP amplitude became significantly larger from parietal to central to fronto-central to frontal sites only for the left and the right electrodes (all ps <0.050), whereas for the midline electrodes, where ERP amplitude reached the highest values, fronto-central and frontal sites did not differ from central sites, but were significantly higher than parietal sites (all ps <0.001).

#### 1200–2000 ms

Mean ERP amplitude extracted in this time window at frontal, fronto-central, central, and parietal electrode sites was analyzed. The ANOVA confirmed that higher ERP amplitude was found for the GS condition [main effect of stimulus size: F(1,21) = 28.08, p<0.001; η^2^_p_ = 0.57]. A significant main effect of anterior-posterior electrode position [F(2,20) = 10.28, p<0.001; η^2^_p_ = 0.33] showed that ERP in such time window were larger at frontal, fronto-central and central sites compared to parietal positions (all ps<0.030). A significant main effect of left-right electrode position [F(2,20) = 12.47, p<0.001; η^2^_p_ = 0.37] revealed that mean ERP amplitude within the 1200–2000 ms time-window was maximal at midline compared to both left and right sites (all ps<0.002).

### Correlations between Kinematic and ERP Events

**Figure 5:**
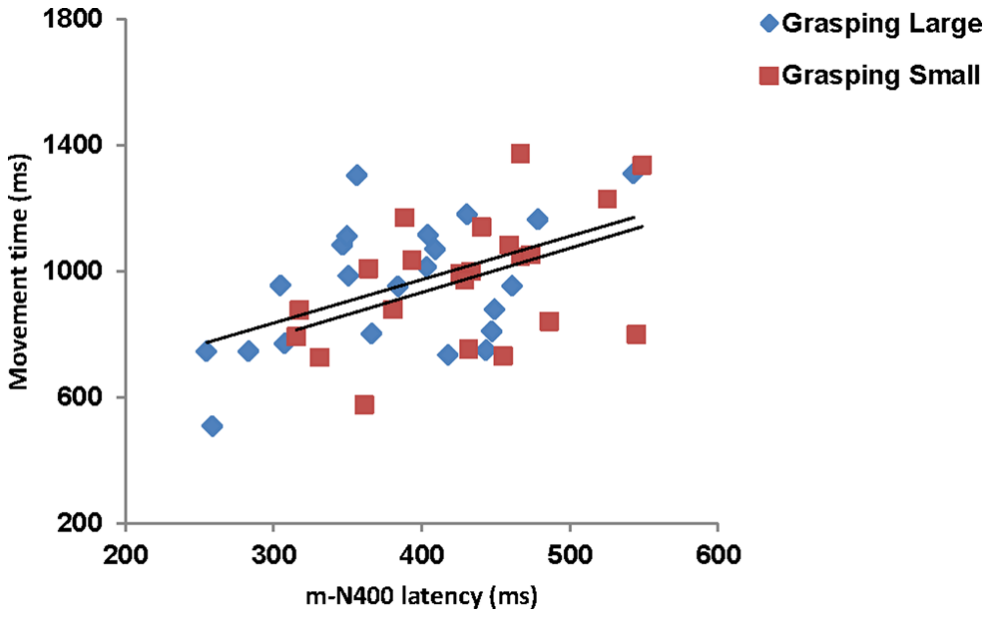
Correlation between movement time and ERP measures. Correlation between the individual data for movement time and the m-N400 latency for the Grasping Large and the Grasping Small conditions.

Mean amplitude and latency of P300 and m-N400 components were averaged at electrode sites in which they were maximally expressed. Specifically, at P3 and Pz for the P300 and at Fz and FCz for the m-N400. Then these values were correlated with movement duration and the considered kinematic measures, namely time to peak velocity and the time of maximum grip aperture. No significant correlations were detected when considering the relationship between kinematical and ERP events. However, as depicted in Figure 5, for both the GL and the GS experimental conditions, the individual mean latency for the m-N400 component significantly correlated with the individual mean for movement time [for GL: r(22) = 0.49, p = 0.022; for GS: r(22) = .46, p = 0.034].

## Discussion

We set out to investigate kinematics and ERP activity during reach-to-grasp movements performed towards either a large or a small stimulus. Overall the results indicate that the two grasping conditions determine a modulation in timing and amplitude of specific kinematic landmarks and ERP components.

In terms of behavioural performance, our results are in line with previous kinematical studies [2], [3], [5], [64]–[66]. Literature findings consistently indicate that, with respect to whole hand grips, precision ones are characterized by a longer movement duration, and an anticipated and lowered amplitude of maximum grip aperture (e.g. [2]–[5], [64]). Customarily, no differences in the times to peak wrist velocity regardless of the type of grasp are usually found. In the same way, here the reaching component was characterized by a bell-shaped wrist velocity profiles with single peaks with no differences in the latency of these peaks depending on grasp type. The total duration of reach-to-grasp movements was longer and the time and amplitude of maximum grip aperture was earlier and smaller for the GS than for the GL condition. These findings indicate that the size of the stimulus influenced selectively the planning and the execution of the reach-to-grasp movement. This is an important aspect of the present study because in order to ascertain the effects that such differential processing might have on ERPs, it is necessary to demonstrate that the participants’ movement show classic kinematic signatures depending on grasp conditions.

For an efficient grasp visual information regarding an object’s physical properties (e.g., size) must be transformed and used to select an appropriate motor command. For both humans and monkeys the key cortical circuit involved in this transformation involves the anterior intraparietal area (AIP), the ventral and dorsal premotor cortices (PMv and PMd, respectively), and the primary motor cortex (M1) [6], [34], [38], [49], [59], [66]–[70]. AIP contains neurons that discharge in relation to specific object properties [68], whereas many grasp-related “canonical” neurons [59] are found in PMv [67], [69], [70]. Experiments in monkey [67], [68] and humans (for review see [9]) appear to show that object properties are encoded as a gradient along the AIP-PMv-M1 axis, with the object being first represented in visual attributes and then in terms of an appropriate grasp.

With this in mind, our EEG recording revealed differences between grip types in the ERPs evoked by stimulus appearance. Such differences were concerned to, both visuo-spatial processing and motor planning. At first, differences in amplitude between conditions become evident over parietal sites following object appearance, at around 300 ms (P300), that is during the planning phase. Such difference remained significantly distinct during the execution phase up to 800 ms. In particular, the peak amplitude for the P300 component was higher for the GL than for the GS condition. This finding might reflect the greater amount of visuo-spatial information to be extracted from larger objects. In this view, object metric properties, such as size, are processed at parietal level. Although we cannot firmly determine the brain source of such activation, it is likely that it reflects AIP activity concerned with the amount of visual information related to the object, and it might be seen as the initial step of the transformation leading from representation of objects to movement. These findings fit with those reported in a recent study on the role of anterior intraparietal sulcus in sensorimotor integration of visually guided hand movements [71]. Here it was shown that the suppression of alpha oscillation over the parieto-occipital electrodes occurred at 220–240 ms following object presentation. Furthermore, the evidence of a parietal involvement is in agreement with neurophysiological evidence showing that parieto-occipital neurons are sensitive to grip type [46], [47]. Assuming that our present results reflect this kind of activity, they might provide a further confirmation that motor plans requiring hand preshaping extend farther anteriorly into both the precuneus and the middle intraparietal sulcus [30]. In the light of previous ERPs studies looking at brain sources of motor-related potentials [52] it is likely to suggest the involvement of additional areas, such as the superior parietal lobe.

Subsequent to the parietal activation we found a negative electrical activity, peaking at around 400 ms following object appearance (m-N400), which was evident over central and frontal electrode sites. The amplitude of this component was higher for the GS than the GL conditions and such difference was significant within a time-window lasting from 400 ms up to 800 ms following object presentation. The polarity, the slow temporal trend and the scalp distribution suggest that such component reflects motor planning and that it is linked to premotor activity [72].

Unlike previous ERP studies on motor planning (e.g. [51]–[53], [73]–[75]), we did not analyze self-paced movements and we did not adopt a precuing task, but we examined EEG deflections evoked by stimulus appearance which prompted a spontaneous grip movement. Nevertheless, the spatio-temporal characteristics of the m-N400 might be assimilated to an index of motor planning and it is strongly influenced by motor variables. Interestingly, stimulus size significantly affected the latency and the topographical location of such component. The m-N400 peak had a later onset and a wider fronto-central distribution for the GS than for the GL condition. This suggests that the planning phase needed for a precision grip movement takes longer and involves more (dorsal) areas. Taken together, these findings indicate that the parietal’ visual information, encoded in an “object” reference frame, is subsequently multiplexed into a “grasp” reference frame within a premotor network possibly involving both the PMd and the PMv. Beyond forming a critical node in the visuomotor planning circuit underlying grasping, recent evidence suggests that different premotor areas (e.g., PMd and PMv) might have dissociable processes. Experiments in humans and monkeys indicate that PMv is more involved in the distal components of the action, such as hand preshaping and specific grip responses (e.g. [41]). PMd, instead, appears to be more involved in the on-line control of movement (e.g. [15], [38]). Given that most of these previous descriptions are based on the characterization of activity stemming during the movement itself, the decoding of different planned hand movements shown here provides a significant additional dimension to such descriptions, which fits with previous functional imaging reports (e.g. [30]).

The difference in amplitude between the GS and the GL condition may reflect the need for additional sensory-motor control mechanisms for the more accurate GS condition. A result that is in keeping with the evidence that accuracy has the ability to affect readiness potentials [72]. In humans, evidence from developmental, psychophysical, neuropsychological and neuroimaging studies seems to suggest that precision grips (as for our GS condition) are characterised by a greater degree of complexity. Firstly, the ability to perform independent finger movements and grasp with the precision grip is not present when voluntary grasping emerges (e.g. [76]). Secondly, consistent results within the adult reach-to-grasp behavioral literature [6], and those obtained in the present study, indicate that the performance of a precision grip is characterized by the need for additional time. Berthier et al. [77] also showed that as visual information and object size decreased, subjects had longer movement times, slower speeds, and more asymmetrical hand-speed profiles. This kinematic characterization reflects the adoption of a strategy following the principles of the Fitt’s Law [78], implying that the difficulty of the task is reflected in movement kinematics. Thirdly, in macaques, it has been revealed that of the premotor area F5 neurons active during grasping, the most frequent were those involved in precision grips [16] whereas whole hand neurons were encountered much less frequently [16], [66]. Finally, neuroimaging studies indicate that premotor activity increase more during the execution of a movement toward a small object than toward a large one reflecting the increased planning and on-line control required by grasping small objects [39], [49]. An alternative possibility related to the modulation of the N-400 activity might be concerned with inhibition. Previous studies from Kok et al [79], demonstrated a frontal N400 elicited by a No-Go stimulus in a Go\No-Go paradigm. In this view the greater N-400 activity for the condition in which a precision grip is performed would stem from inhibiting the opening of the whole hands in order to specify index finger and thumb when a precise grasping is requested. This idea would imply that the ‘simpler’ whole hand grasp would be prepared by default and then precision grip would be specified. Evidence that this process might be in place comes from reach-to-grasp perturbation studies in which the passage from whole hand to precision grip movements has been measured [80].

Altogether, the present findings confirm a parietal processing related to the vision of a particular graspable object which provides premotor cortices with grasp-related information that allows neurons in these areas to be tuned to the upcoming grasp and on-line control. Importantly, they provide an addition to current literature by revealing the time course of the visuomotor transformation process starting from the ‘parietal’ visual object discrimination activity to the ‘premotor’ activity concerned with the assignment of specific hand configurations depending on object’s size. Furthermore, they show that once such differential process, depending on grasp type/stimulus size ensemble within the ‘parietal’ and the ‘frontal’ component of the grasping circuit is started before movement initiation, it remains sustained throughout the entire action. This indicates that these areas participate in a sensorimotor network involved in grasp planning, prediction of sensory stimulation, and monitoring of appropriate execution of the desired actions. And also suggest the role of the parietal (possibly the AIP region) and premotor cortices (possibly PMv and PMd) not only during the execution of reaching-to-grasp movements as previously reported [81]–[82], but also during the planning phase. A result which is in line with neurophysiological evidence showing that the discharge of F5 neurons is tuned for specific grasps well before movement onset and this early tuning was carried over in the preshaping period of the task. In line with this evidence we found a marked differentiation across different grasps during the premovement phase which was carried over into early grasp phases (as witnessed by kinematical analysis) characterized by a premotor kind of activity. Altogether, these properties are consistent with the notion that premotor areas play a role in translating visual information about an object’s physical properties into the appropriate motor plans to interact with the same object [16], [67], [69], [83].

Another aspect of the present findings is concerned with some relationship between kinematical and ERPs events. Of interest is that the ERPs differences noticed during the planning phase at both parietal and premotor level depending on grasp conditions persisted all along the unfolding of the action. And remained statistically different at the time key kinematic landmarks such as the time of maximum grip aperture occurred. A greater peak of maximum grip aperture and a modulation of the time occurrence for this peak corresponded to a significantly different level of activity for ERPs components. This signifies that when the stimulus become visually available sensory and motor processes specifically tailored to process the stimulus were established and maintained active as to organize the kinematical unfolding of the movement.

Although we did not find any significant correlation between the times at which peak ERPs components and the considered kinematical landmarks occurred, we found that for both the GL and the GS conditions the individual mean latency for the premotor m-N400 component significantly correlated with the individual mean for movement time. This might indicate that at the time the ‘parietal’ information regarding the visual aspect of the object are integrated within the premotor area (possibly PMv) with the motor prototypes adequate to successfully grasp it, the time to perform the action is kept into account. Similarly, an estimate of movement time, possibly performed at the level of PMd, might serve to plan the amount of on-line control required by the movement. This mode of programming might keep the timing of the commands independent from the spatial parameters of the movement. In other words, selection of the muscles needing to be activated to carry out a given task can be modified, or the kinematics can be modulated within a centrally generated temporal template that determines the co-ordination of a given action. This might appear to be the easiest and most readily chosen organizational option of the neural system to compensate for the postural and joint kinematic variability characterizing reach-to-grasp actions.

### Conclusions

In the present study, we have explored the kinematic and ERP dynamics during a reach-to-grasp task. Together, kinematical and ERPs data confirm that the object size/type of grasp ensemble has the ability to modulate both the behavioural and the neural components underlying this kind of action. Analysis of the changes at the level of the ERPs components revealed that the parieto-frontal network is modulated differently by prehension movements towards differently sized objects at both planning and execution level. The correlation between movement time and ERPs components is suggestive of a mode of programming relying on a centrally generate template within which dynamic aspects of the movement are coordinated. In a broader perspective, this work underlines the use of EEG for the investigation of movements with unique cortical motor processes such as reach-to-grasp movements.

## References

[1] Jeannerod M (1981) Intersegmental coordination during reaching at natural visual objects. In: Long J, Baddley A, editors. Attention and Performances IX. Hillsdale, NJ: Erlbaum;153–168.

[2] Jeannerod M (1984) The timing of natural prehension movements. J Mot Behav 16: 235–254. 15151851 10.1080/00222895.1984.10735319

[3] Gentilucci M, Castiello U, Corradini ML, Scarpa M, Umiltà C (1991) Influence of different types of grasping on the transport component of prehension movements. Neuropsychologia 29: 361–378. 1886680 10.1016/0028-3932(91)90025-4

[4] Chieffi S, Gentilucci M (1993) Coordination between the transport and the grasp components during prehension movements. Exp Brain Res 94: 471–477. 8359261 10.1007/BF00230205

[5] Jakobson LS, Goodale MA (1991) Factors affecting higher-order movement planning: a kinematic analysis of human prehension. Exp Brain Res 86: 199–208. 1756790 10.1007/BF00231054

[6] Castiello U (2005) The neuroscience of grasping. Nat Rev Neurosci 6: 726–736. 16100518 10.1038/nrn1744

[7] Culham JC, Valyear KF (2006) Human parietal cortex in action. Curr Opin Neurobiol 16: 205–212. 16563735 10.1016/j.conb.2006.03.005

[8] Brochier T, Umiltà MA (2007) Cortical control of grasp in non-human primates. Curr Opin Neurobiol 17: 637–643. 18294839 10.1016/j.conb.2007.12.002

[9] Castiello U, Begliomini C (2008) The cortical control of visually guided grasping. Neuroscientist 14: 157–170. 18219055 10.1177/1073858407312080

[10] Filimon F (2010) Human cortical control of hand movements: parietofrontal networks for reaching, grasping, and pointing. Neuroscientist 16: 388–407. 20817917 10.1177/1073858410375468

[11] Grafton ST (2010) The cognitive neuroscience of prehension: recent developments. Exp Brain Res 204: 475–491. 20532487 10.1007/s00221-010-2315-2 PMC2903689

[12] Godschalk M, Lemon RN, Nijs HG, Kuypers HG (1981) Behaviour of neurons in monkey peri-arcuate and precentral cortex before and during visually guided arm and hand movements. Exp Brain Res 44: 113–116. 7274360 10.1007/BF00238755

[13] Moll L, Kuypers HG (1977) Premotor cortical ablations in monkeys: contralateral changes in visually guided reaching behavior. Science 198: 317–319. 410103 10.1126/science.410103

[14] Passingham RE (1987) Two cortical systems for directing movement. Ciba Found Symp 132: 151–164. 3322713 10.1002/9780470513545.ch10

[15] Raos V, Umiltá MA, Gallese V, Fogassi L (2004) Functional properties of grasping-related neurons in the dorsal premotor area F2 of the macaque monkey. J Neurophysiol 92: 1990–2002. 15163668 10.1152/jn.00154.2004

[16] Rizzolatti G, Camarda R, Fogassi L, Gentilucci M, Luppino G, et al (1988) Functional organization of inferior area 6 in the macaque monkey. II. Area F5 and the control of distal movements. Exp Brain Res 71: 491–507. 3416965 10.1007/BF00248742

[17] Weinrich M, Wise SP (1982) The premotor cortex of the monkey. J Neurosci 2: 1329–1345. 7119878 10.1523/JNEUROSCI.02-09-01329.1982 PMC6564318

[18] Taira M, Mine S, Georgopoulos AP, Murata A, Sakata H (1990) Parietal cortex neurons of the monkey related to the visual guidance of hand movement. Exp Brain Res 83: 29–36. 2073947 10.1007/BF00232190

[19] Fagg AH, Arbib M (1998) Modeling parietal-premotor interactions in primate control of grasping. Neural Networks 11: 1277–1303. 12662750 10.1016/s0893-6080(98)00047-1

[20] Kalaska JF, Scott SH, Cisek P, Sergio LE (1997) Cortical control of reaching movements. Curr Opin Neurobiol 7: 849–859. 9464979 10.1016/s0959-4388(97)80146-8

[21] Fattori P, Gamberini M, Kutz DF, Galletti C (2001) ‘Arm reaching’neuron in the parietal area V6A of the macaque monkey. Eur J Neurosci 13: 2309–2313. 11454035 10.1046/j.0953-816x.2001.01618.x

[22] Buneo CA, Jarvis MR, Batista AP, Andersen RA (2002) Direct visuomotor transformations for reaching. Nature 416: 632–636. 11948351 10.1038/416632a

[23] Battaglia-Mayer A, Caminiti R, Lacquaniti F, Zago M (2003) Multiple levels of representation of reaching in the parieto-frontal network. Cereb Cortex 13: 1009–1022. 12967918 10.1093/cercor/13.10.1009

[24] Gregoriou GG, Savaki HE (2003) When vision guides movement: a functional imaging study of the monkey brain. NeuroImage 19: 959–967. 12880824 10.1016/s1053-8119(03)00176-9

[25] Cavina-Pratesi C, Goodale M, Culham JC (2007) FMRI reveals a dissociation between grasping and perceiving the size of real 3D objects. PLoS One 2: e424. 17487272 10.1371/journal.pone.0000424 PMC1855433

[26] Króliczak G, Cavina-Pratesi C, Goodman D, Culham JC (2007) What does the brain do when you fake it? An FMRI study of pantomimed and real grasping. J Neurophysiol 97: 2410–2422. 17229828 10.1152/jn.00778.2006

[27] Culham JC, Cavina-Pratesi C, Singhal A (2006) The role of parietal cortex in visuomotor control: what have we learned from neuroimaging? Neuropsychologia 44: 2668–2684. 16337974 10.1016/j.neuropsychologia.2005.11.003

[28] Tunik E, Rice NJ, Hamilton A, Grafton ST (2007) Beyond grasping: representation of action in human anterior intraparietal sulcus. Neuroimage 36: T77–86. 17499173 10.1016/j.neuroimage.2007.03.026 PMC1978063

[29] Olivier E, Davare M, Andres M, Fadiga L (2007) Precision grasping in humans: from motor control to cognition. Curr Opin Neurobiol 17: 644–648. 18337084 10.1016/j.conb.2008.01.008

[30] Gallivan JP, McLean DA, Valyear KF, Pettypiece CE, Culham JC (2011) Decoding action intentions from preparatory brain activity in human parieto-frontal networks. J Neurosci 31: 9599–9610. 21715625 10.1523/JNEUROSCI.0080-11.2011 PMC6623162

[31] Grafton ST, Fagg H, Woods RP, Arbib M (1996) Functional anatomy of pointing and grasping in humans. Cereb Cortex 6: 226–237. 8670653 10.1093/cercor/6.2.226

[32] Faillenot I, Toni I, Decety J, Grégoire MC, Jeannerod M (1997) Visual pathways for object-oriented action and object recognition: functional anatomy with PET. Cereb Cortex 7: 77–85. 9023435 10.1093/cercor/7.1.77

[33] Dohle C, Posse S, Stephan KM, Hefter H, et al (1998) Human anterior intraparietal area subserves prehension: a combined lesion and functional MRI activation study. Neurology 50: 1253–1259. 9595971 10.1212/wnl.50.5.1253

[34] Ehrsson HH, Fagergren A, Jonsson T, Westling G, Roland S, et al (2000) Cortical Activity in Precision-Versus Power-Grip Tasks:An fMRI Study. J Neurophysiol 83: 528–536. 10634893 10.1152/jn.2000.83.1.528

[35] Ehrsson HH, Kuhtz-Buschbeck JP, Forssberg H (2002) Brain regions controlling nonsynergistic versus synergistic movement of the digits: a functional magnetic resonance imaging study. J Neurosci 22: 5074–5080. 12077202 10.1523/JNEUROSCI.22-12-05074.2002 PMC6757747

[36] Culham JC, Danckert SL, DeSouza JFX, Gati JS, Menon RS, et al (2003) Visually guided grasping produces fMRI activation in dorsal but not ventral stream brain areas. Exp Brain Res 153: 180–189. 12961051 10.1007/s00221-003-1591-5

[37] Frey SH, Vinton D, Norlund R, Grafton ST (2005) Cortical topography of human anterior intraparietal cortex active during visually guided grasping. Cogn Brain Res 23: 397–405. 10.1016/j.cogbrainres.2004.11.010 15820646

[38] Begliomini C, Caria A, Grodd W, Castiello U (2007) Comparing natural and constrained movements: new insights into the visuomotor control of grasping. PloS One 2: e1108. 17971871 10.1371/journal.pone.0001108 PMC2040199

[39] Begliomini C, Nelini C, Caria A, Grodd W, Castiello U (2008) Cortical activations in humans grasp-related areas depend on hand used and handedness. PloS One 3: e3388. 18846222 10.1371/journal.pone.0003388 PMC2561002

[40] Davare M, Andres M, Clerget E, Thonnard JL, Olivier E (2007) Temporal dissociation between hand shaping and grip force scaling in the anterior intraparietal area. J Neurosci 27: 3974–3980. 17428971 10.1523/JNEUROSCI.0426-07.2007 PMC6672552

[41] Davare M, Andres M, Cosnard G, Thonnard JL, Olivier E (2006) Dissociating the role of ventral and dorsal premotor cortex in precision grasping. J Neurosci 26: 2260–2268. 16495453 10.1523/JNEUROSCI.3386-05.2006 PMC6674806

[42] Chapman H, Gavrilescu M, Wang H, Kean M, Egan G, et al (2002) Posterior parietal cortex control of reach-to-grasp movements in humans. Eur J Neurosci 15: 2037–2042. 12099909 10.1046/j.1460-9568.2002.02021.x

[43] Connolly JD, Andersen RA, Goodale MA (2003) FMRI evidence for a ‘parietal reach region’ in the human brain. Exp Brain Res 153: 140–145. 12955383 10.1007/s00221-003-1587-1

[44] Prado J, Clavagnier S, Otzenberger H, Scheiber C, Kennedy H, et al (2005) Two cortical systems for reaching in central and peripheral vision. Neuron 48: 849–858. 16337921 10.1016/j.neuron.2005.10.010

[45] Cavina-Pratesi C, Monaco S, Fattori P, Galletti C, McAdam TD, et al (2010) Functional magnetic resonance imaging reveals the neural substrates of arm transport and grip formation in reach-to-grasp actions in humans. J Neurosci 30: 10306–10323. 20685975 10.1523/JNEUROSCI.2023-10.2010 PMC6634677

[46] Fattori P, Breveglieri R, Marzocchi N, Filippini D, Bosco A, et al (2009) Hand orientation during rieach-to-grap movements modulates neuronal activity in the medial posterior parietal area V6A. J Neurosci 29: 1928–1936. 19211899 10.1523/JNEUROSCI.4998-08.2009 PMC6666273

[47] Fattori P, Raos V, Breveglieri R, Bosco A, Marzocchi N, et al (2010) The dorsomedial pathway is not just for reaching: grasping neurons in the medial parieto-occipital cortex of the macaque monkey. J Neurosci 30: 342–349. 20053915 10.1523/JNEUROSCI.3800-09.2010 PMC6632536

[48] Battaglini PP, Muzur A, Galletti C, Skrap M, Brovelli A, et al (2002) Effects of lesions to area V6A in monkeys. Exp Brain Res 144: 419–422. 12021823 10.1007/s00221-002-1099-4

[49] Grol MJ, Majdandzić J, Stephan KE, Verhagen L, Dijkerman HC, et al (2007) Parieto-frontal connectivity during visually guided grasping. J Neurosci 27: 11877–11887. 17978028 10.1523/JNEUROSCI.3923-07.2007 PMC2703728

[50] Wheaton LA, Yakota S, Hallett M (2005) Posterior parietal negativity preceding self-paced praxis movements. Exp Brain Res 163: 535–539. 15883800 10.1007/s00221-005-2314-x

[51] Wheaton LA, Shibasaki H, Hallett M (2005) Temporal activation pattern of parietal and premotor areas related to praxis movements. Clin Neurophysiol 116: 1201–1212. 15826863 10.1016/j.clinph.2005.01.001

[52] Bozzacchi C, Giusti MA, Pitzalis S, Spinelli D, Di Russo F (2012) Awareness affects motor planning for goal-oriented actions. Biol Psychol 89: 503–514. 22234365 10.1016/j.biopsycho.2011.12.020

[53] Zaepffel M, Brochier T (2012) Planning of visually guided reach-to-grasp movements: inference from reaction time and contingent negative variation (CNV). Psychophysiology 49: 17–30. 21895686 10.1111/j.1469-8986.2011.01277.x

[54] Walter WG (1964) Slow potential waves in the human brain associated with the expectancy, attention and decision. Arch für Psychiatr Nervenkr 206: 309–322. 14345314

[55] Loveless NE, Sanford AJ (1974) Slow potential correlates of preparatory set. Biol Psychol 1: 303–314. 4425714 10.1016/0301-0511(74)90005-2

[56] Gaillard AW (1977) The late CNV wave: preparation versus expectancy. Psychophysiology 14: 563–568. 928607 10.1111/j.1469-8986.1977.tb01200.x

[57] Rohrbaugh J, Gaillard AWK (1983) Sensory and motor aspects of the contingent negative variation. In Gaillard AWK, Ritter W, editors. Tutorials in event-related potential research: Endogenous components 269–310.

[58] Leuthold H, Sommer W, Ulrich R (2004) Preparing for action: inference from CNV and LRP. Psychophysiology 18: 77–88.

[59] Rizzolatti G, Luppino G (2001) The cortical motor system. Neuron 31: 889–901. 11580891 10.1016/s0896-6273(01)00423-8

[60] Oldfield RC (1971) The assessment and analysis of handedness: the Edinburgh inventory. Neuropsychologia 9: 97–113. 5146491 10.1016/0028-3932(71)90067-4

[61] Delorme A, Makeig S (2004) EEGLAB: an open source toolbox for analysis of single-trial EEG dynamics. J Neuroscie Met 134: 9–21. 10.1016/j.jneumeth.2003.10.009 15102499

[62] Jung TP, Makeig S, Humphries C, Lee TW, McKeown MJ, et al (2000) Removing electroencephalographic artifacts by blind source separation. Psychophysiology 37: 163–178. 10731767

[63] Jung TP, Makeig S, Westerfield M, Townsend J, Courchesne E, et al (2000) Removal of eye activity artifacts from visual event-related potentials in normal and clinical subjects. Clin Neurophysiol 111: 1745–1758. 11018488 10.1016/s1388-2457(00)00386-2

[64] Castiello U (1996) Grasping a fruit: selection for action. J Exp Psychol Hum Percept Perform 22: 582–603. 8666954 10.1037//0096-1523.22.3.582

[65] Smeets JB, Brenner EA (1999) New view on grasping. Motor Control 3: 237–271. 10409797 10.1123/mcj.3.3.237

[66] Jeannerod M, Arbib MA, Rizzolatti G, Sakata H (1995) Grasping objects: the cortical mechanisms of visuomotor transformation. Trends Neurosci 18: 314–320. 7571012

[67] Raos V, Umiltá MA, Murata A, Fogassi L, Gallese V (2006) Functional properties of grasping-related neurons in the ventral premotor area F5 of the macaque monkey. J Neurophysiol 95: 709–729. 16251265 10.1152/jn.00463.2005

[68] Murata A, Gallese V, Luppino G, Kaseda M, Sakata H (2000) Selectivity for the shape, size, and orientation of objects for grasping in neurons of monkey parietal area AIP. J Neurophysiol 83: 2580–2601. 10805659 10.1152/jn.2000.83.5.2580

[69] Murata A, Fadiga L, Fogassi L, Gallese V, Raos V, et al (1997) Object representation in the ventral premotor cortex (area F5) of the monkey. J Neurophysiol 78: 2226–2230. 9325390 10.1152/jn.1997.78.4.2226

[70] Umilta MA, Brochier T, Spinks RL, Lemon RN (2007) Simultaneous recording of macaque premotor and primary motor cortex neuronal populations reveals different functional contributions to visuomotor grasp. J Neurophysiol 98: 488–501. 17329624 10.1152/jn.01094.2006

[71] Verhagen L, Dijkerman HC, Medendorp WP, Toni I (2012) Cortical dynamics of sensorimotor integration during grasp planning. J Neurosci 32: 4508–4519. 22457498 10.1523/JNEUROSCI.5451-11.2012 PMC6622056

[72] Shibasaki H, Hallett M (2006) What is the Bereitschaftspotential? Clin Neurophysiol 17: 2341–2356. 10.1016/j.clinph.2006.04.025 16876476

[73] Kourtis D, Sebanz N, Knoblich G (2012) EEG correlates of Fitts’s law during preparation for action. Psychol Res 76: 514–524. 22311155 10.1007/s00426-012-0418-z PMC3383955

[74] Leuthold H, Jentzsch I (2009) Planning of rapid aiming movements and the contingent negative variation: are movement duration and extent specified independently? Psychophysiology 46: 539–550. 19496231 10.1111/j.1469-8986.2009.00799.x

[75] Müller-Gethmann H, Rinkenauer G, Stahl J, Ulrich R (2000) Preparation of response force and movement direction: onset effects on the lateralized readiness potential. Psychophysiology 37: 507–514. 10934909

[76] Gordon AM, Forssberg H, Iwasaki N (1994) Formation and lateralization of internal representations underlying motor commands during precision grip. Neuropsychologia 32: 555–568. 8084414 10.1016/0028-3932(94)90144-9

[77] Berthier NE, Clifton RK, Gullapalli V, McCall DD, Robin DJ (1996) Visual Information and object size in the control of reaching. J Mot Behav 28: 187–197. 12529202 10.1080/00222895.1996.9941744

[78] Fitt PM (1954) The information capacity of the human motor system in controlling the amplitude of movement. J Exp Psychol Hum Percept Perform 47: 381–391. 13174710

[79] Kok A (1986) Effects of degradation of visual stimulation on components on the event-related potential (ERP) in go/nogo reaction tasks. Biol Psychol 23: 21–38. 3790646 10.1016/0301-0511(86)90087-6

[80] Castiello U, Bennett KM, Stelmach GE (1993) Reach to grasp: the natural response to object size. Exp Brain Res 94: 163–178. 8335072 10.1007/BF00230479

[81] Rice NJ, Tunik E, Grafton ST (2006) The anterior intraparietal sulcus mediates grasp execution, independent of requirement to update: new insights from transcranial magnetic stimulation. J Neurosci 26: 8176–8182. 16885231 10.1523/JNEUROSCI.1641-06.2006 PMC6673775

[82] Xiao J, Padoa-Schioppa C, Bizzi E (2006) Neuronal correlates of movement dynamics in the dorsal and ventral premotor area in the monkey. Exp Brain Res 168: 106–119. 16177830 10.1007/s00221-005-0074-2

[83] Brochier T, Spinks RL, Umilta MA, Lemon RN (2004) Patterns of muscle activity underlying object-specific grasp by the macaque monkey. J Neurophysiol 92: 1770–1782. 15163676 10.1152/jn.00976.2003

