# Correction: Co-Registering Kinematics and Evoked Related Potentials during Visually Guided Reach-to-Grasp Movements

**DOI:** 10.1371/annotation/07033e55-8e0f-42f9-9729-13331551444a

**Published:** 2013-11-06

**Authors:** Teresa De Sanctis, Vincenza Tarantino, Elisa Straulino, Chiara Begliomini, Umberto Castiello

There was an error in the figure legend of Figure 4. Please see the corrected Figure 4 here: 

**Figure:**
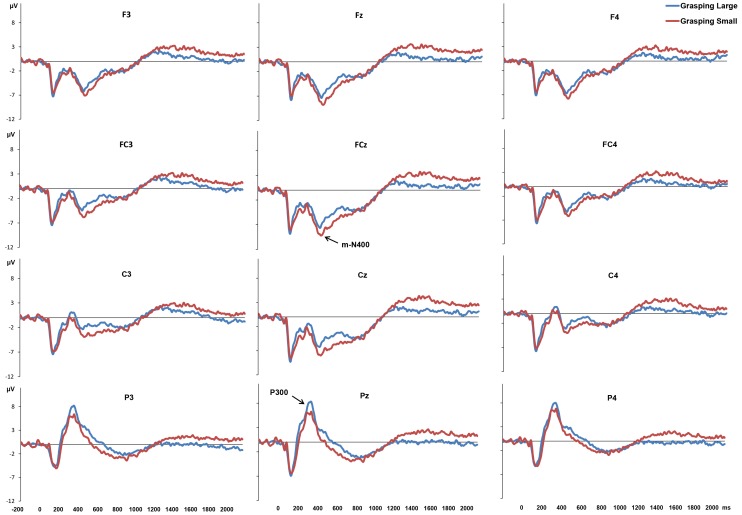


The corrected Figure 4 caption reads:

Grand-average ERP waveforms for the Grasping Small and the Grasping large conditions. The plots show ERPs time locked to glasses opening.

The corrected Figure 3 caption reads:

Scalp distribution of ERP differences between Grasping Small and Grasping Large conditions.

In the last sentence of the "Electrophysiological recording and data processing" section in Materials and Methods, some of the stimulus appearance sites were listed incorrectly. The corrected sentence is:

 Based on visual inspection of grand average waveforms and amplitude scalp maps, the following ERP components were statistically analyzed: amplitude and latency of P300, namely the positive peak evoked 200–400 ms following stimulus appearance at parietal sites (P3, Pz, P4); amplitude and latency of N400, namely the negative peak occurring at 300–500 ms after object appearance at frontal (F3, Fz, F4), fronto-central (FC4, FCz, FC3), and central (C3, Cz, C4) sites; and mean amplitude of the sustained negativity observed in 400–800 and 1200–2000 time windows at frontal (F3, Fz, F4), fronto-central (FC4, FCz, FC3), central (C3, Cz, C4), and parietal (P3, Pz, P4) sites.

